# Novel *SCYL2* Mutations and Arthrogryposis Multiplex Congenita 4: Case Report and Review of the Literature

**DOI:** 10.3390/ijms26073079

**Published:** 2025-03-27

**Authors:** Khaled Zamel, Abdulrahman Ahmed Al-Subaiey, Mohamed Alsabbagh, Abeer Fadda, Amira Saeed, Bruno Mourao Pacheco, Bernice Lo, Ruba Benini

**Affiliations:** 1 Division of Pediatric Neurology, Department of Pediatrics, Sidra Medicine, Doha 26999, Qatar; khz3001@qatar-med.cornell.edu; 2 Department of Clinical Pediatrics, Weill-Cornell Medical College, Doha 24144, Qatar; brunomouraopac@gmail.com; 3 Research Department, Sidra Medicine, Doha 26999, Qatar; aalsubaiey@sidra.org (A.A.A.-S.); malsabbagh@sidra.org (M.A.); faddaabeer@gmail.com (A.F.); amirasaeed1967@gmail.com (A.S.); blo@sidra.org (B.L.); 4 College of Health and Life Sciences, Hamad Bin Khalifa University, Doha 34110, Qatar

**Keywords:** SCYL2, arthrogryposis, neurodevelopmental disorder

## Abstract

SCY1 Like Pseudokinase 2 (SCYL2) is a protein that regulates secretory protein trafficking and plays a pivotal role in neurodevelopment by attenuating excitotoxicity. Neurogenic arthrogryposis due to *SCYL2* mutations, also known as arthrogryposis multiplex congenita 4 (AMC4), is a rare condition that presents with microcephaly, agenesis of the corpus callosum, optic atrophy, global developmental delay, and early lethality. We used whole-exome sequencing to identify pathogenic variants, DynaMut2 to determine the predicted effect on protein stability, and Western blot to investigate the effect on protein expression. We present two novel missense mutations in *SCYL2* resulting in loss of function at the protein level in a pediatric case of AMC4, further highlighting the key role of SCYL2 in neuronal cell survival and healthy brain development. There is diversity in the pathological features among previously published cases of AMC4, most likely due to the nature of each mutation. This report summarizes the clinical data of all known patients with *SCYL2* mutations.

## 1. Introduction

Neurodevelopmental disorders (NDDs) are a diverse group of disorders that affect central nervous system development and function, and they present with various genetic associations and phenotypic symptoms. NDDs typically arise from an interference in essential neurodevelopmental processes that are tightly controlled by a complex interplay of gene expression and environmental challenges. Consequently, NDDs have a multifaceted origin with more than 800 disease-associated genes reported [1]. Clinically, NDDs can lead to an inability to reach cognitive, emotional, or motor developmental milestones. They include disorders such as autism spectrum disorder, intellectual disability, attention deficit hyperactivity disorder, and epilepsy, among others [2,3]. Moreover, NDDs comprise a serious health burden, affecting more than 3% of children worldwide [4].

Identification of NDD-causing genes is essential for providing genetic counseling and elucidating the molecular pathways affected to implement targeted therapies. Several genetic aberrations have been identified in NDDs as causative events, such as chromosomal rearrangements, copy number variations, small indels, and point mutations [5]. However, direct genotype–phenotype correlations in NDDs remain elusive due to the existence of multiple genetic factors, such as gene vulnerability and mutational load, as well as environmental factors that influence disease manifestation.

In recent years, functional and molecular studies have begun to elucidate how different mutations disturb converging molecular pathways leading to the identification of potential therapeutic targets [5]. Familial NDDs are particularly useful for delineating the contribution of genetic and nongenetic factors to the pathogenesis of these disorders in the presence of a shared genetic background. Moreover, many polygenic NDDs have recently been shown to be the result of a culmination of numerous common genetic variants and the epistatic, or functional, interaction between these genes [6].

One of the key molecular pathways affected in NDDs is the synaptic signaling pathway, which is crucial for neurodevelopment. The activity-dependent pruning of neuronal circuits during development depends on cell-adhesion molecules, which are responsible for organizing the pre- and postsynaptic compartments through trans-cellular signaling, and synaptic signaling-associated proteins, mainly comprised of receptors at the postsynaptic density [7,8,9].

SCYL2 is an ATP-binding, serine/threonine pseudokinase that is expressed in clathrin-coated vesicles. In rodents, SCYL2 has been shown to play a crucial role in brain development by preventing excitotoxicity in the hippocampus. Loss of *Scyl2* causes perinatal lethality and loss of major neuronal populations and sensorimotor deficits in surviving mice [10]. *SCYL2* mutations in humans have only been described in two reports previously. The first report described two unrelated families from consanguineous parents with homozygous truncating mutations, presenting with arthrogryposis multiplex and agenesis of the corpus callosum in a novel disorder termed Zain syndrome or AMC4 [11]. The second recently published report identified one set of patients with a loss-of-function mutation and a phenotype that recapitulates some of the aspects of AMC4, as well as another set of patients with missense mutations with a much milder phenotype mainly consisting of autism spectrum disorder [12]. In this case report, we describe the clinical presentation of an infant with novel homozygous mutations in SCYL2 with microcephaly, arthrogryposis multiplex, optic atrophy, epilepsy, recurrent infections, autonomic dysfunction, and agenesis of the corpus callosum.

## 2. Case Presentation

### 2.1. Case Description

**Figure 1:**
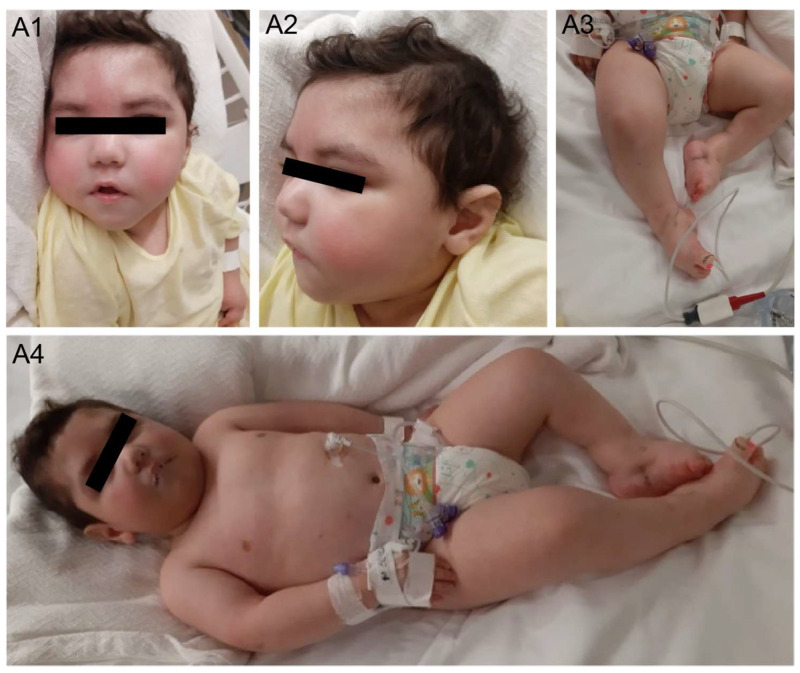
Clinical images of patient: (**A1**,**A2**) facial dysmorphic features including prominent forehead, micrognathia, bulbous nose, small mouth, and abnormal ear folding; (**A3**,**A4**) arthrogryposis of both upper and lower limbs.

The male proband was the second live birth from consanguineous Egyptian parents. The mother was on enoxaparin during the pregnancy due to a history of three previous miscarriages. The pregnancy was otherwise uneventful, and the child was born at term via caesarian section for maternal reasons (uterine scar). Birth weight was 2600 g (10th percentile). There was no previous relevant family history, and his sister was in good health. Physical examination noted at birth included dysmorphic facial features, microcephaly, bilateral talipes equinovarus deformities, and poor sucking prompting admission to the neonatal intensive care unit for two weeks (Figure 1).

In the first few months of life, he underwent casting for correction of the bilateral talipes equinovarus deformities. Starting from the age of 6 months, he had multiple hospital admissions for recurrent upper respiratory tract infections, necessitating intensive care. He was found to have dysphagia and was at increased risk for aspiration. Nasogastric tube feeding was initiated at the age of 6 months and eventually underwent Nissen fundoplication and gastric tube insertion. In addition to his dysphagia, he was found to have a profound global developmental delay with poor head control, inability to roll, and not cooing. At the age of 15 months, he presented with a febrile status epilepticus lasting for one hour, and Levetiracetam was initiated. An electroencephalogram showed generalized slowing but no epileptic abnormalities. He subsequently developed unprovoked breakthrough seizures occurring every two months, characterized by generalized tonic stiffening and necessitating optimization of the antiseizure treatment. At the age of 18 months, his physical examination was significant for severe microcephaly (43.3 cm, <2%, z-score −3.15), facial dysmorphisms, talipes equinovarus deformities, multiple mild contractures, decreased spontaneous antigravity movements, axial hypotonia, diminished deep tendon reflexes, and 3–4 beat clonus at the ankles bilaterally (Figure 1). Between 18 months and 20 months of age, he continued to have repeated admissions for gastroenteritis or chest infections and febrile seizures and at least two documented neutropenic episodes. A detailed review of his infectious episodes revealed three documented viral infections (RSV and parainfluenza 4 twice), with most respiratory admissions attributed to suspected viral upper respiratory tract infections and aspiration pneumonias. The patient also displayed features of dysautonomia, including episodes of unexplained tachycardia, hypotension, aseptic fevers with negative infectious workup, and episodes of unexplained vomiting. He was ultimately admitted with a severe illness with respiratory distress but no seizures and died at the age of 22 months.

**Figure 2:**
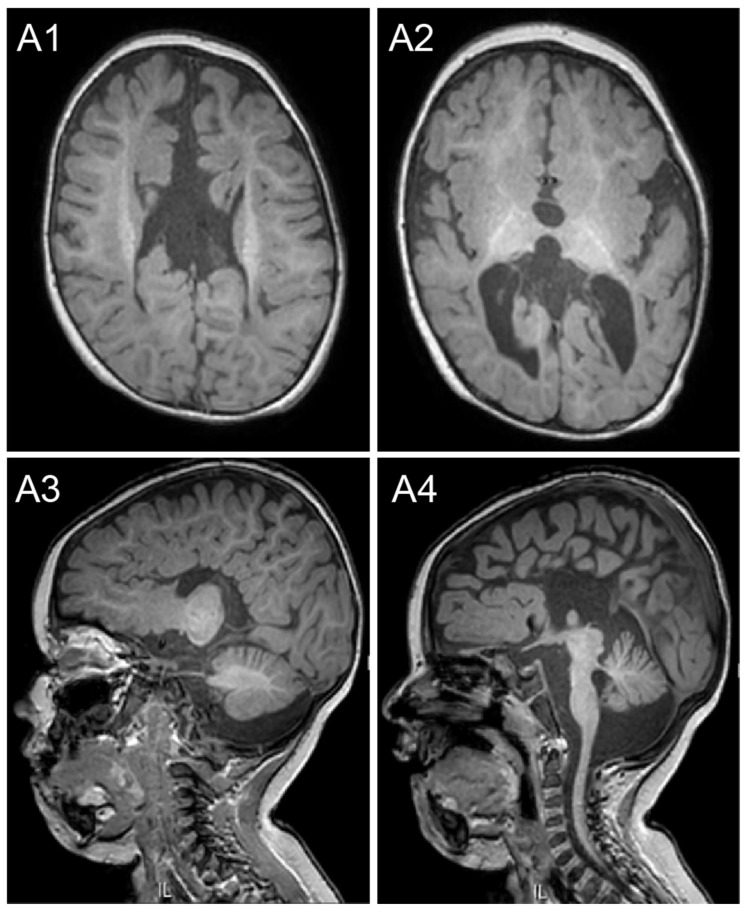
MRI brain images showing cranio-facial disproportionality (microcephaly); complete corpus callosum agenesis with enlarged ventricles (bilateral colpocephaly), reduced white matter volume, and disproportionate ponto-cerebellar volume reduction: (**A1**,**A2**) T1 axial sequences; (**A3**,**A4**) T1 sagittal sequences.

Ophthalmologic evaluation revealed bilateral optic atrophy and mild esotropia. Cardiac evaluation including a cardiac echo was unremarkable. Magnetic resonance imaging (MRI) conducted at the age of 13 months showed multiple abnormalities, including agenesis of the corpus callosum, ponto-cerebellar volume reduction, and non-specific white matter abnormalities (Figure 2).

### 2.2. Genetic Variants

**Figure 3:**
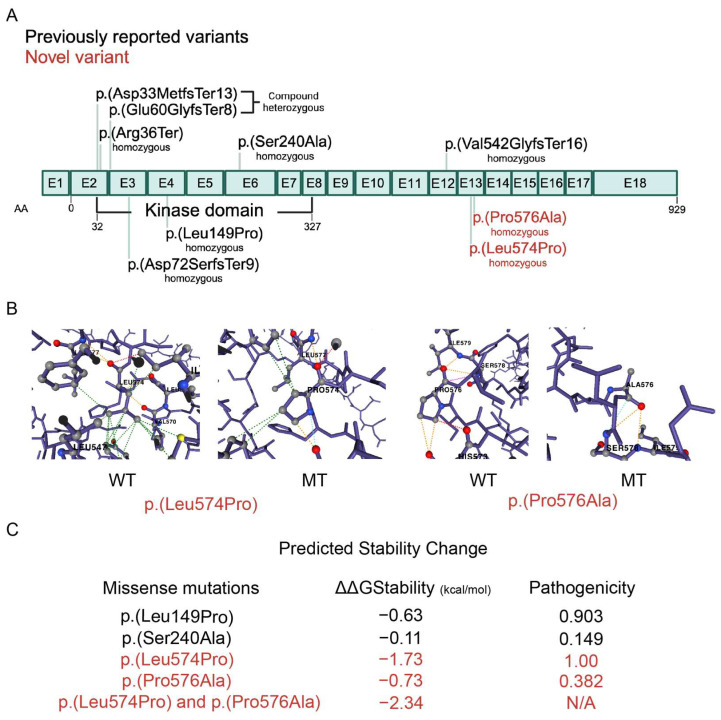
SCYL2 amino acid mutations. (**A**) Schematic showing exons of *SCYL2* (NM_017988.6) with all reported protein variants (NP_060458.3). Previously reported variants in black. Novel variants from this report in red. (**B**) Cartoon model showing hydrophobic interactions (green), polar interactions (orange), and hydrogen bonds (red). The p.(Leu574Pro) mutant loses 11 hydrophobic interactions and 1 polar interaction in comparison to the wildtype, and the p.(Pro576Ala) mutant loses 2 polar interactions and 1 hydrogen bond in comparison to the wildtype. (**C**) Dynamut2 predicted stability changes and AlphaMissense predicted pathogenicity for all missense mutations (0 = benign, 1 = pathogenic).

With whole-exome sequencing, potential pathogenic CNVs were not found. However, several homozygous variants predicted to be damaging were present; this is not surprising given the consanguinity of the family. WES revealed two novel homozygous adjacent variants in the *SCYL2* gene inherited from both parents (Supplementary Figure S1), classified as variants of uncertain significance at that point (Table 1), as well as three very rare mutations never reported in the homozygous state (Table 2). The novel variant c.1721T>C changes leucine 574 to proline (p.L574P), and the variant c.1726C>G changes proline 576 to alanine (p.P576A) in SCYL2 (Figure 3A). These variants are predicted to be deleterious by several in silico pathogenicity prediction tools, including SIFT, PolyPhen-2, MutationTaster, and CADD. Furthermore, the predicted stability change for the two novel mutations were found to be −1.73 and −0.73 kcal/mol, respectively, with a combined stability change of −2.34 kcal/mol (Figure 3C).

### 2.3. Protein Expression

**Figure 4:**
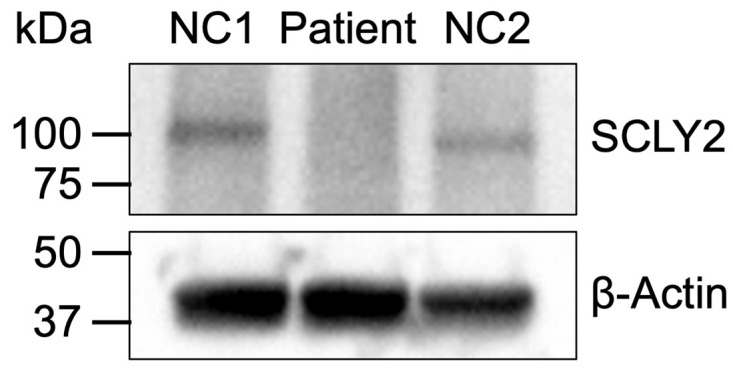
Western blot of SCYL2 (expected size of 104 kDa) and β-actin protein from T cells of patient and non-related healthy donors (NC1 and NC2).

To determine the impact of these variants on SCYL2 protein expression, a Western blot was performed using lysates from T cells expanded from patient and unrelated healthy donor PBMCs. SCYL2 protein levels in the patient were undetectable compared to healthy donors (Figure 4). This expression loss indicated that the variant is likely pathogenic and impacts SCYL2 protein expression or stability, which supports the predicted reduced stability of the protein harboring both mutations (Figure 3C).

## 3. Discussion

We present a case of an infant with homozygous damaging mutations in *SCYL2* and a severe neurodevelopmental disorder characterized by microcephaly, arthrogryposis multiplex, optic atrophy, epilepsy, recurrent infections, autonomic dysfunction (recurrent sterile febrile illness, unexplained tachycardia, intermittent hypotension, unexplained vomiting, and constipation), and congenital brain anomalies including agenesis of the corpus callosum, ponto-cerebellar volume reduction, and non-specific white matter abnormalities. Although nerve conduction studies were not performed, the diminished deep tendon reflexes suggested the presence of an underlying peripheral neuropathy, similar to the patients in the first report of AMC4 as well as patients with *SCYL1* loss of function [11,13].

Previous studies have shown that the SCYL family of pseudokinases are essential in preventing neuronal degeneration in mice [10,14]. Murine knockout of *Scyl2* resulted in rapid postnatal death due to their inability to feed properly, reminiscent of the dysphagia observed in our patient. The small fraction of *Scyl2^−/−^* mice that did survive exhibited growth delay and sensorimotor deficits. Neuron-specific conditional deletion of *SCYL2* resulted in a recapitulation of the phenotype observed in the global knockout, with a slightly reduced severity of symptoms, indicating a neuronal phenotypic origin. Moreover, the neuron-specific *Scyl2^−/−^* mice displayed reduced brain size and loss of CA3 pyramidal neurons, which are known for their role in memory formation as well as their susceptibility to neurodegeneration and seizures [15]. This loss was found to be the result of a degenerative process that started between postnatal day 14.5 and 21.5, with an increase in activated glial cells, a marker of neuroinflammation.

Excitotoxic cell death is an apoptosis-mediated process that is triggered in neurons by overactivation of calcium-permeable glutamate receptors [16]. Excitotoxicity is the mechanism by which neuronal loss occurs in many neurodegenerative disorders, and CA3 neurons are particularly sensitive to excitotoxity [17]. There are several observations that support the role of SCYL2 as protective against excitotoxicity. For example, the loss of CA3 neurons in neuron-specific *Scyl2^−/−^* mice coincided with the period of mossy fiber-CA3 synapse formation between postnatal day 4.5 and 8.5 [10]. In addition, the degeneration of CA3 neurons in these knockout mice exhibited characteristics of apoptosis, and lastly, c-Fos, a marker of excitotoxic cell death [18], was highly upregulated in the CA3 neurons leading up to their loss.

SCYL2 colocalizes and interacts with clathrin and its adaptor proteins AP1 and AP3 [10,19]. Intriguingly, however, clathrin-mediated functions such as transferrin and EGF endocytosis, EGFR internalization, and sorting of lysosomal enzymes, which are all impaired in clathrin-, AP1-, AP2-, or AP3-depeleted cells, are intact in *Scyl2^−/−^* mouse embryonic fibroblasts (MEFs). The ESCORT-0 complex, composed of the proteins HRS, STAM1, or STAM2, is important in the multivesicular body pathway, which is involved in targeting ubiquitinated membrane proteins for lysosomal degradation. Upon knockout of *Hrs* in neurons, the development of severe neurological defects concomitant with a loss of CA3 neurons was observed [20]. This similarity in phenotype between the *Hrs* knockout and *Scyl2* knockout suggested a shared pathway. However, unlike HRS, which targets EGFR to the lysosome, *Scyl2*-deficient MEFs did not exhibit any differences in EGFR turnover [10]. These findings suggest that SCYL2 functions independently of clathrin and conventional lysosomal pathways in its regulation of excitatory receptor trafficking. SCYL2 may play a role in protein quality control, like the role of SCYL1 in the ER–Golgi intermediate compartment in COPI vesicles [21].

In humans, recessive mutations in *SCYL1* resulting in complete loss of the protein were identified in two unrelated families with a similar syndrome characterized by liver failure, peripheral neuropathy, cerebellar atrophy, and ataxia [13]. Fibroblasts isolated from patients with *SCYL1* loss-of-function mutations displayed an enlarged Golgi apparatus, which is consistent with SCYL1’s function in retrograde vesicle trafficking from the Golgi to the ER [21,22], as failure of this transport is known to cause abnormal Golgi morphology [23].

Disruptions in COPI- and COPII-mediated signaling results in Golgi apparatus fragmentation, as seen in animal models of amyotrophic lateral sclerosis and progressive motor neuropathy mice that lack the Golgi-localized tubulin-binding cofactor E (TBCE) [24,25]. Dysfunction of the Golgi apparatus has been associated with several neurodegenerative diseases, in particular in motor neuropathies [26]. Motor neurons can reach up to 1 m in length and are known to have a large Golgi apparatus network that extends into the dendrites and axon, potentially making them more vulnerable to disruptions in Golgi apparatus function than other cell types. Fibroblasts isolated from patients with spinal muscular atrophy (SMA), where lower motor neurons degenerate, displayed abnormal Golgi apparatus morphology. Similarly, depletion of the survival of motor neuron protein (SMN) in motor neuron-like cells resulted in a similar abnormal Golgi apparatus morphology as well as defective transport of Golgi-resident proteins. This abnormality was rescued upon expression of COP1 coatomer subunit alpha-COP or SMN [27]. Given the link between proper Golgi apparatus structure and function and motor neuron survival, it is plausible that the dysautonomia, as part of the motor neuron disease observed in our patient, is linked to SCYL2’s function in the trans-Golgi network [19].

Additionally, SCYL2 was found to be upregulated during the antiviral type I interferon response and was also shown to limit viral particle release through the recruitment of protein phosphatase 2A (PP2A). PP2A was found to dephosphorylate viral protein U (Vpu), a human immunodeficiency virus-1 accessory protein, preventing it from degrading tetherin, which plays an important role in restricting viral progeny release from infected cells [28,29]. This antiviral feature of SCYL2, and its dysfunction in our patient, may have played a role in the recurrent upper respiratory tract infections observed, although infections were not reported in any of the other patients with *SCYL2* mutations [11,12].

Mutations in SCYL2 result in arthrogryposis multiplex congenita 4, neurogenic, with agenesis of the corpus callosum (OMIM# 616365), reported for the first time in 2020 in two families. The novel homozygous truncating mutation [NM_017988.6:c.106C>T: p.(Arg36Ter)] was identified in an 8-month-old infant, born of consanguineous Saudi parents with a similarly affected sibling and two affected second-degree cousins. The second homozygous truncating frameshift mutation [NM_017988.6:c.1624dupG:p.(Val542GlyfsTer16)] was identified in a 6-year-old girl from an Omani family, also consanguineous with history of miscarriages, and three similarly affected family members. Altogether constituting eight patients, this novel autosomal recessive phenotype presented with severe peripheral and central nervous system involvement with microcephaly, colpocephaly, dilated brain ventricles, seizures, optic atrophy, dysmorphic features, agenesis of corpus callosum, global developmental delay, arthrogryposis multiplex, fixed flexion joint deformity, undescended testes, feeding difficulty, severe neutropenia, and death at early age [11].

The second report of *SCYL2* mutations in humans was published recently with a variety of symptoms, further expanding the phenotypic spectrum of SCYL2-associated disorders. A total of five novel mutations were identified: three bi-allelic loss-of-function variants in two cases and two bi-allelic missense variants of unknown significance in two cases. The two cases with loss-of-function mutations presented with arthrogryposis with corpus callosum agenesis; however, they were negative for some of the other symptoms reported in the first case report, such as ventriculomegaly, hydramnios, microcephaly, seizures, optic atrophy, and pathological fractures. The other two cases with missense variants presented with a much milder phenotype consisting of speech delay, autism spectrum disorder, and some morphological features such as synophrys and lop undifferentiated ears in one case and brachycephaly and clinodactyly in the other case. These findings could be indicative of a broad phenotypic spectrum of SCYL2-related disorders depending on which part of the protein is affected. However, the two cases with mild symptoms also presented with other variants of uncertain significance that could contribute to the phenotype: a hemizygous maternally inherited missense variant in *FLNA* in one case and a de novo heterozygous frameshift variant of uncertain significance reported in *RBPJ* (Table 1).

Our patient’s pathological symptoms most closely resemble the patients reported in the first case report, who displayed more severe symptoms and experienced an early death (Table 1). Moreover, our mutations appear in an exon adjacent to where one of the first mutations was identified (Figure 3A). Since we have confirmed that SCYL2 is undetectable at the protein level in our patient (Figure 4), we hypothesize that the severity of symptoms might be associated with protein expression levels and stability. This is further supported by the increased predicted instability of SCYL2 in our patient, which is −2.34 kcal/mol, compared to the two previously reported missense mutations that are only associated with a mild phenotype consisting of developmental delay, which have stability changes of −0.63 and −0.11 kcal/mol, respectively (Figure 3C). However, further studies are necessary to definitively determine whether the inability to detect the protein is due to instability or other factors such as ubiquitination and degradation.

The combination of recurrent viral infections and aspiration pneumonias in our patient may reflect dual pathogenic mechanisms: impaired antiviral responses due to SCYL2’s role in viral particle release regulation and increased aspiration risk due to bulbar dysfunction. The presence of dysautonomia extends our understanding of SCYL2’s role beyond the central nervous system, suggesting its importance in peripheral nervous system function through the regulation of vesicular trafficking in autonomic neurons. This observation aligns with the broader neurological phenotype seen in *Scyl2*^−/−^ mice and suggests that SCYL2-related disorders may represent a spectrum of central and peripheral nervous system involvement.

## 4. Materials and Methods

### 4.1. Sample Collection and Processing

The family was consented and recruited into a research study approved by the Institutional Review Board (IRB #1500762) at Sidra Medicine. Blood was drawn from the proband as well as from both parents and sent for whole-exome sequencing (WES) at Sidra Genomics Core. DNA was isolated using a DNeasy Blood & Tissue Kit (Qiagen, Hilden, Germany) following the manufacturer’s recommendations. DNA quantity and quality were checked using a Nanodrop spectrophotometer (Thermo Fisher Scientific, Waltham, MA, USA). DNA processed for the WES was subjected to Illumina HiSeq sequencing, generating 150 bp paired-end reads with an average of 70 million reads and >50× coverage. 

### 4.2. Data Processing and Analysis

Reads were aligned with a bowtie to GRCh37 reference genome with more than 99% of reads aligned. SNPs and indels were called with GATK; CNVs were detected with NxClinical software v5.1 (BioDiscovery, Hawthorne, CA, USA). Pathogenic variant analysis was conducted with NxClinical software v5.1. CNV overlapping segments in the Database of Genomic Variants (DGV) or those that are common in our internal database of Middle Eastern subjects were excluded. Filters for SNP indels were applied: read depth > 20, MAPQ  >  30, base quality > 20, minor allele frequency (MAF)  <  0.01 (1%), and in silico prediction of pathogenicity by SIFT and/or PolyPhen. The GRCh38 reference genome was used for the chromosomal locations of the variants in the tables.

### 4.3. Dynamut2 Analysis

A predicted protein structure file was downloaded from AlphaFold [30] (File AF-Q6P3W7-F1-v4) and used to generate a consensus prediction of different mutations on SCYL2 protein stability using DynaMut2 [31]. Stability changes were reported in Gibbs Free Energy (ΔΔ*G*). Pathogenicity was determined using the AlphaMissense Pathogenicity Heatmap, where 0 indicates a benign variant and 1 indicates a pathogenic variant (Figure 3) “https://alphafold.ebi.ac.uk/entry/Q6P3W7 (accessed on 24 November 2024)”.

### 4.4. Western Blot Analysis

Peripheral blood mononuclear cells (PBMCs) from the patient and healthy donors were isolated from whole blood using Ficoll-Hypaque gradient separation. T cells were activated and expanded by culturing PBMCs with antibodies to CD3 (1 mg/mL) and CD28 (1 mg/mL) in complete Advanced RPMI medium for 3 days then expanded in the presence of 100 U/mL IL-2 for at least 3 days. Cells were then lysed with a lysis buffer containing 30 mM Tris-HCl pH 7.4, 120 mM NaCl, 2 mM KCl, 3 mM EDTA, 1% Triton X-100, and 1× Halt Protease Inhibitor Cocktail (Thermo Fisher Scientific, Waltham, MA, USA). The proteins were resolved using SDS-PAGE then transferred onto a PVDF membrane. The membrane was blocked with 5% milk in TBS-T then probed for SCYL2 using an antibody to SCYL2 (Santa Cruz Biotechnology, Dallas, TX, USA #515916). A representative blot from three independent experiments is shown (Figure 4).

## 5. Conclusions

This study demonstrates the similarity between our patient and previously reported *SCYL2* loss-of-function patients with AMC4, further supporting the role of SCYL2 in central and peripheral nervous system development and suggesting an additional function in antiviral responses. Moreover, our finding highlights the need for the identification and the study of additional SCYL2-deficient patients, with the use of proteomic and functional investigations to further clarify genotype–phenotype relationships, especially *SCYL2* variant impact on symptom severity and lethality. Future studies elucidating the pathomechanism of SCYL2 mutations are also needed to provide insight into potential targeted treatments for SCYL2-deficient patients.

## Figures and Tables

**Table 1:** Summary of all known *SCYL2* mutations and associated clinical findings.

Type of Variant	Bi-Allelic Missense Variants			Bi-Allelic Loss-of-Function Variants			
Cases	Case 1	Case 2	Case 3	Case 4	Case 5	Cases 6–9	Cases 10–11
Gender	Male	Male	Male	Male	Male	3 males and 1 female	2 females
Arthrogryposis	Yes	No	No	Yes	Yes	Yes (4/4)	Yes (2/2)
Corpus callosum agenesis	Yes	No	Not available	Yes	Yes	Yes (4/4)	Yes (2/2)
Cerebral findings	Pronto-cerebellar volume reduction and white matter abnormalities	No	Not available	Polymicrogyria in the insula, agenesis of the posterior corpus callosum, and pyramidal tract hypoplasia	Cerebral and cerebellar cortical atrophy	Frontoparietal cortical atrophy (3/3)	No (2/2)
Psychomotor delay	Yes, severe global developmental delay affecting motor, language, and social/communicative spheres	Yes, speech delay and poor understanding but normal motor development	Yes, speech delay, particularly for expressive language with a better receptive language	Not applicable	Yes	Yes (4/4)	Yes (2/2)
Ventriculomegaly	Yes	No	Not available	No	No	Yes (2/3)	Yes (2/2)
Reduced fetal movements	Yes	No	No	Yes	No	Yes (4/4)	Yes (2/2)
Polyhydramnios	No	No	No	No	No	Yes (4/4)	Yes (2/2)
Microcephaly	Yes	No	Yes <1st percentile	No	No	Yes (2/3)	Yes (2/2)
Cryptorchidism	No	Yes, unilateral	No	Testicles not present in the scrotum (at 27 weeks of gestational age)	Yes	Yes (3/3)	Not applicable
Micrognathia	Yes	Yes	No	Yes	No	Yes (4/4)	Yes (2/2)
Optic atrophy	Yes, bilateral with mild esotropia	No	No	Not available	No	Yes (3/3)	Yes (1/1)
Spasticity	Yes	No	No	Not applicable	No	Yes (4/4)	Yes (2/2)
Seizures	Febrile status epilepticus and epilepsy	No	No	Not applicable	No	Yes (3/3)	Yes (1/1)
Other morphological features	Prominent forehead, depressed nasal bridge, bulbous nose, low-set abnormally folded ears, small mouth, thin lips, and short neck	Synophrys, mild slant eyes, lop undifferentiated ears, and thick lower lip	Brachycephaly, low anterior hairline, heavy eyebrows, long eyelashes, large upper central incisors, clinodactyly, mild 2–3–4 finger syndactyly, and narrow 5th toenails	Square forehead, depressed nasal bridge, broad nasal tip, low-set slightly dysplastic ears, hypoplastic helix, thin lips, and short neck	No	Prominent forehead (4/4), depressed nasal bridge (3/3), bulbous nose (4/4), low-set abnormally folded ears (4/4), small mouth (4/4), thin lips (4/4), and short neck (4/4)	Prominent forehead (2/2), depressed nasal bridge (2/2), bulbous nose (2/2), low-set abnormally folded ears (2/2), small mouth (2/2), thin lips (2/2), and short neck (1/2)
Pathological fractures	No	No	No	No	No	Yes (3/4)	Yes (2/2)
Other signs	Dysphagia, gastroenteritis, chest infections, febrile seizures, neutropenic episodes, and died at 22 months	Some findings of autism spectrum disorder and unilateral inguinal hernia repair	Autism spectrum disorder	Mild calf muscle hypoplasia	Inguinal hernia and bilateral hip dysplasia	Feeding difficulties (4/4) and death between 8 and 30 months (4/4)	Feeding difficulties (2/2) and death at 3 years (1/2)
Variant (GRCh38)	chr12: 100329279T>C and chr12: 100329284C>G	chr12: g.100298141T>C	chr12: g.100312519T>G	chr12: g.100283067del and chr12: g.100283146dup	chr12: g.100291539_100291559delinsTC	chr12: g.100283076C>T	chr12: g.100326736dup
Variant (NM_017988.6)	c.1721T>C and c.1726C>G	c.446T>C	c.718T>G	c.97del and c.176dup	c.214_234delinsTC	c.106C>T	c.1624dup
Amino acid variant	p.(Leu574Pro) and p.(Pro576Ala)	p.(Leu149Pro)	p.(Ser240Ala)	p.(Asp33MetfsTer13) and p.(Glu60GlyfsTer8)	p.(Asp72SerfsTer9)	p.(Arg36Ter)	p.(Val542GlyfsTer16)
Inheritance	Both parents heterozygous	Both parents heterozygous	Both parents heterozygous	Paternal and maternal, respectively	Both parents heterozygous	Both parents heterozygous	Both parents heterozygous
Classification	Uncertain significance	Uncertain significance	Uncertain significance	Both pathogenic	Pathogenic	Pathogenic	Pathogenic
ACMG criteria and REVEL score (if applicable)	c.1721T>C PM2 PP3 REVEL score: 0.850 c.1726C>G PM2 REVEL score: 0.202	PM2 PP3 REVEL score: 0.918	PM2 REVEL score: 0.205	Both PVS1 PM2 PM3	PVS1 PM2 PM3	PVS1 PM2 PM3	PVS1 PM2 PM3
Other variants	See Table 2	FLNA: NM_001110556.2: c.5975G>C p.(Gly1992Ala) Hemizygous VUS	RBPJ: NM_005349.4: c.173del p. (Lys58SerfsTer48) Heterozygous De novo VUS	No	No	Not available	Not available

This table has been adapted from Malbos et al. [12] license ID 1590527-1; VUS, variant of uncertain significance.

**Table 2:** Summary of other rare and novel mutations identified.

Genes	Transcript ID	HGVSp	HGVSc	Genotype	Pop. Allele Freq %	PolyPhen Prediction	SIFT Prediction	dbSNP	Chromosome Region	Varsome
SCN5A	NM_198056.2	p.(Val1202Met)	c.3604G>A	Hom	0.006	Probably damaging	Deleterious	rs375509048	chr3:38,575,359	VUS
KLHL40	NM_152393.3	p.(Val452Ala)	c.1355T>C	Hom	0.065	Benign	Deleterious	rs140389534	chr3:42,686,651	VUS
STAB1	NM_015136.2	p.(Pro1978Ser)	c.5932C>T	Hom	0.003	Probably damaging	Deleterious	rs752987636	chr3:52,521,384	Likely pathogenic

Hom, homozygous; Pop, population.

## Data Availability

Data are contained within the article.

## Supplementary Materials

The following supporting information can be downloaded at https://www.mdpi.com/article/10.3390/ijms26073079/s1.

## Abbreviations

The following abbreviations are used in this manuscript:

NDDS	Neurodevelopmental disorders
SCYL2	SCY1-like 2
CNS	Central nervous system
WES	Whole-exome sequencing
IRB	Institutional review board
PBMCs	Peripheral blood mononuclear cells
RPMI	Roswell Park Memorial Institute (refers to cell culture medium)
TBST	Tris-buffered saline with tween
PVDF	Polyvinylidene fluoride
CNVs	Copy number variations
SNPs	Single-nucleotide polymorphisms
DGV	Database of genomic variants
MAF	Minor allele frequency
SIFT	Sorting intolerant from tolerant (pathogenicity prediction tool)
GRCh37	Genome reference consortium human build 37
GRCh38	Genome reference consortium human build 38
SDS-PAGE	Sodium dodecyl sulfate polyacrylamide gel electrophoresis
EGFR	Epidermal growth factor receptor
MEFs	Mouse embryonic fibroblasts
ESCORT-0	Endosomal sorting complex required for transport-0
CA3	Cornu Ammonis 3 (region of the hippocampus)
OMIM	Online Mendelian inheritance in man
CADD	Combined annotation dependent depletion
