# Supplementary material for: Novel SCYL2 Mutations and Arthrogryposis Multiplex Congenita 4: Case Report and Review of the Literature
Source: Int J Mol Sci. 2025 Mar 27;26(7):3079. doi: 10.3390/ijms26073079 (PMC11988787; doi:10.3390/ijms26073079)
Supplement: Supplementary file 1 [file ijms-26-03079-s001.zip › ijms-3512748-supplementary.pdf]

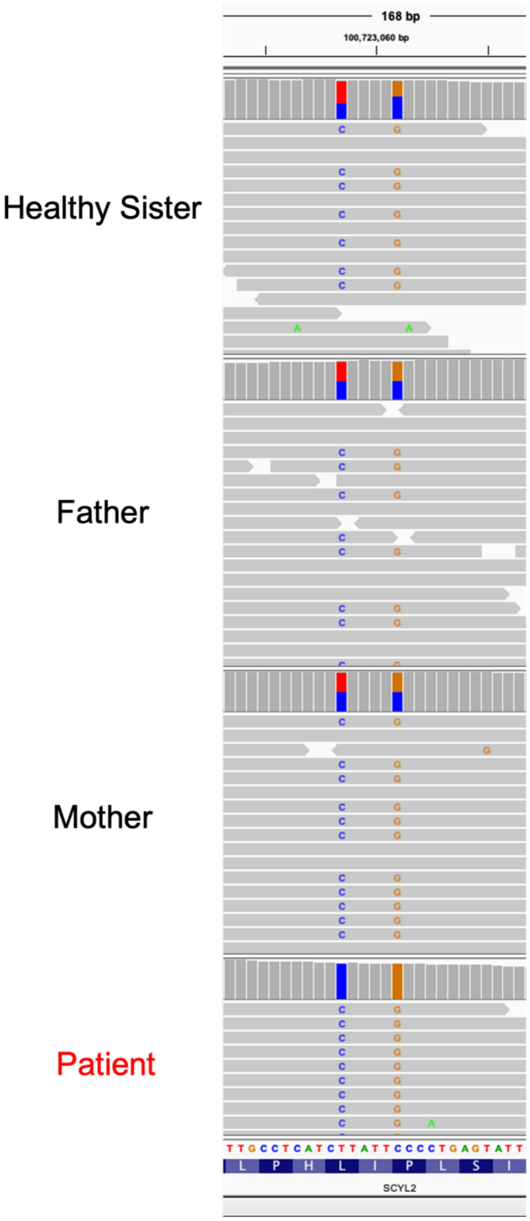

Supplementary Figure 1: WES read alignment showing the *SCYL2* variants in the patient and healthy family members. Alignment performed on Human GRCh37/hg19 reference genome.
